# Hole Transfer Layer Engineering for CdTe Nanocrystal Photovoltaics with Improved Efficiency

**DOI:** 10.3390/nano10071348

**Published:** 2020-07-10

**Authors:** Yasi Jiang, Yiyang Pan, Wanhua Wu, Kaiying Luo, Zhitao Rong, Sihang Xie, Wencai Zuo, Jingya Yu, Ruibo Zhang, Donghuan Qin, Wei Xu, Dan Wang, Lintao Hou

**Affiliations:** 1 School of Materials Science and Engineering, South China University of Technology, Guangzhou 510640, China; 201730321056@mail.scut.edu.cn (Y.J.); 201730321247@mail.scut.edu.cn (Y.P.); 201636291439@mail.scut.edu.cn (W.W.); msluoky@mail.scut.edu.cn (K.L.); 201720117942@mail.scut.edu.cn (Z.R.); 201730321308@mail.scut.edu.cn (S.X.); 201730321407@mail.scut.edu.cn (W.Z.); 201730303175@mail.scut.edu.cn (J.Y.); 201730321377@mail.scut.edu.cn (R.Z.); xuwei@scut.edu.cn (W.X.); wangdan@scut.edu.cn (D.W.); 2 State Key Laboratory of Luminescent Materials & Devices, Institute of Polymer Optoelectronic Materials & Devices, South China University of Technology, Guangzhou 510640, China; 3 Siyuan Laboratory, Guangzhou Key Laboratory of Vacuum Coating Technologies and New Energy Materials, Guangdong Provincial Key Laboratory of Optical Fiber Sensing and Communications, Department of Physics, Jinan University, Guangzhou 510632, China

**Keywords:** CdTe, nanocrystal, solar cells, Spiro

## Abstract

Interface engineering has led to significant progress in solution-processed CdTe nanocrystal (NC) solar cells in recent years. High performance solar cells can be fabricated by introducing a hole transfer layer (HTL) between CdTe and a back contact electrode to reduce carrier recombination by forming interfacial dipole effect at the interface. Here, we report the usage of a commercial product 2,2′,7,7′-tetrakis[N,N-di(4-methoxyphenyl)amino]-9,9′-spirobifluorene (Spiro) as a hole transfer layer to facilitate the hole collecting for CdTe nanocrystal solar cells. It is found that heat treatment on the hole transfer layer has significant influence on the NC solar cells performance. The *J*_sc_, *V*_oc_, and power conversion efficiency (PCE) of NC solar cells are simultaneously increased due to the decreased contact resistance and enhanced built-in electric field. We demonstrate solar cells that achieve a high PCE of 8.34% for solution-processed CdTe NC solar cells with an inverted structure by further optimizing the HTL annealing temperature, which is among the highest value in CdTe NC solar cells with the inverted structure.

## 1. Introduction

Solution-processed nanocrystal (NC) solar cells have demonstrated as a candidate for low cost, environmentally friendly, and large scale “roll to roll” printing product due to their low raw material consumption and simple fabrication process [1,2,3]. Recently, intensive investigations have been focused on CdTe NC solar cells with an inverted structure [4,5]. Comparing to the conventional CdTe NC solar cells using indium tin oxide (ITO) as the anode, devices with an inverted structure have many advantages, such as long-term stability under ambient conditions by avoiding the usage of low work-function metal cathode on the top of the device and better photon absorption efficiency due to the small distance between the active layer and the illumination source [6,7]. Due to the high resistance and low doping concentration of CdTe NC thin film, it is difficult to obtain a low resistance contact between CdTe and the back contact electrode [8]. In order to fabricate efficient CdTe NC solar cells with an inverted structure, matching energy level between the CdTe NC and anode to reduce interfacial recombination is of essential importance to promote solar cell efficiency [9]. Introducing a thin hole transfer layer (HTL) between the CdTe NC and anode has been confirmed to be an effective way to reduce interfacial charge recombination and improve device performance [10]. It is well known that Cu-doped interface layer has been applied successfully to CdTe thin film solar cells prepared by close-space sublimation (CSS) method [11,12]. Unfortunately, fabricating a homogeneous Cu-doped layer on CdTe NC thin films remains a challenge, since Cu may diffuse quickly along the crystal boundaries of NCs and restrict further device performance improvement [13]. Yang et al. [14] investigated the application of commercially available poly-[3-(potassium-6-hexanoate)thiophene-2, 5-diy] (P3KT) as a HTL for aqueous processed CdTe NC solar cells with the configuration of ITO/SnO_2_/CdS/CdTe/P3KT/MoOx/Au and found that the insertion of P3KT optimized bandgap alignment and enhanced carrier collection efficiency. There were several reports about using triphenylamine type polymers such as poly(diphenylsilane-co-4-vinyl-triphenylamine) (Si-TPA) or poly(phenylphosphine-co-4-vinyl-triphenylamine) (P-TPA) as a HTL. In these reports, high power conversion efficiency (PCE) up to 9% is obtained, which is the highest value ever reported for CdTe NC solar cells with an inverted structure [15,16]. Researchers found that strong binding exists between Cd and N atoms and a dipole layer can thus be formed, which effectively decreases interfacial recombination and improves carrier collection efficiency. 

In this paper, we develop a commercially available 2,2′,7,7′-tetrakis[N,N-di(4-methoxyphenyl)amino]-9,9′-spirobifluorene (Spiro) as the HTL for CdTe NC solar cells. Spiro has been widely and successfully applied in perovskite solar cells due to its matchable band alignment, good conductivity, and non-reactivity with active layers [17,18]. Furthermore, additives such as lithium bis(trifluoromethanesulfonyl) imide (TFSI-Li) [19], copper salts cuprous thiocyanate (CuPC) [20], or Co(III)-based complexes [21] can increase the conductance of Spiro and further improve device performance. In the case of CdTe NC solar cells, Yang et al. found that by introducing the Spiro HTL, an interfacial dipole layer was formed, which facilitated charge collection and improves open circuit voltage (*V*_oc_)/PCE compared to devices without Spiro [22]. Although improved performance (with PCE of 6.56%) is obtained for the solar cell with the Spiro HTL in this case, the current density vs voltage (*J–V*) curve shows roll-over phenomena, which imply that large contact resistance exists between the CdTe NC layer and HTL film.

Here, we further study the CdTe NC solar cells with the configuration of ITO/ZnO/CdS/CdSe/CdTe/HTL/Au by using Spiro as an HTL. The usage of gradient electron transport layer modification in the cathode had been confirmed to decrease carrier recombination and improve the light harvesting efficiency in both short and long wavelength regions [23]. It was found that the annealing temperature on the HTL had a significant effect on the NC solar cells performance. The impact of HTL annealing temperature on the solar cell performance was studied by external quantum efficiency (EQE), *J–V*, capacitance−voltage (*C*−*V*) analysis, and transient photovoltage (TPV) measurements. At an optimized annealing temperature, the solar cell with the Spiro HTL had an efficiency of 8.34%, and the *V*_oc_, short circuit current density (*J*_sc_), and fill factor (FF) were 0. 71 V, 23.11 mA/cm^2^, and 50.83%, respectively. On the other hand, the control device delivers an efficiency of 7.08%, and the *V*_oc_, *J*_sc_, and FF of 0.65 V, 21.91 mA/cm^2^, and 49.71%, respectively. Therefore, the NC device incorporating Spiro HTL shows significant and simultaneous improvements in *J*_sc_, *V*_oc_, and FF, which lead to 18% higher PCE than the device without HTL and 28% higher PCE than the similar device reported before.

## 2. Experiment

**Figure 1:**
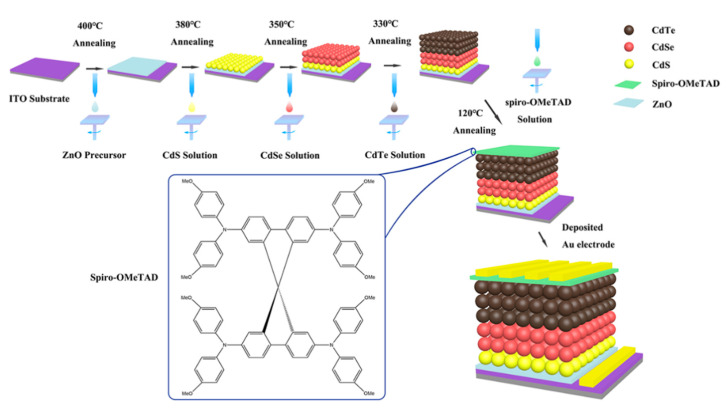
A schematic of the fabrication process of nanocrystal (NC) solar cells.

CdS NCs, CdSe NCs, CdTe NCs, and ZnO precursors were prepared based on previous reported methods [15,24]. Spiro (CAS: 207739-72-8) was purchased from Derthon Optoelectronic Materials Science Technology Co., Ltd. (Shenzhen, China) and used as received. The CdTe NC solar cell with the ITO/ZnO/CdSe/CdTe/Spiro/Au configuration was fabricated by layer-by-layer spin-coating solution process. In a typical process, as shown in Figure 1, the Zn^2+^ precursor solution was firstly deposited on ITO substrates by spin-casting and then annealed at 400 °C for 10 min to eliminate any organic impurities and form the ZnO layer. Then, one layer of CdS NCs, two layers of CdSe NCs, and five layers of CdTe NCs films were deposited on the ZnO film in sequence using a layer-by-layer solution process under ambient conditions, as reported in the literature [16,25]. To prepare solution for the fabrication of HTL, Spiro was dissolved in chlorobenzene with a concentration of 80 mg·mL^−1^ while Li-TFSI was dissolved in acetonitrile with a concentration of 520 mg·mL^−1^. Then, 1 mL of Spiro solution, 18 μL of Li-TFSI solution, and 29 μL of 4-tert-butylpyridine were mixed together to obtain the Li-doped Spiro precursor. Several drops of Li-doped Spiro precursor were then put on top of the CdTe NC thin film and spin-casted at 3000 rpm for 20 s. The above substrates were then annealed at 25~120 °C for 15 min under ambient conditions to improve conductivity and preserved in a desiccator for overnight oxidation of the Spiro [26]. Finally, a gold electrode (80 nm) was deposited via thermal evaporation through a shadow mask with an active area of 0.16 cm^2^.

## 3. Results and Discussion

**Figure 2:**
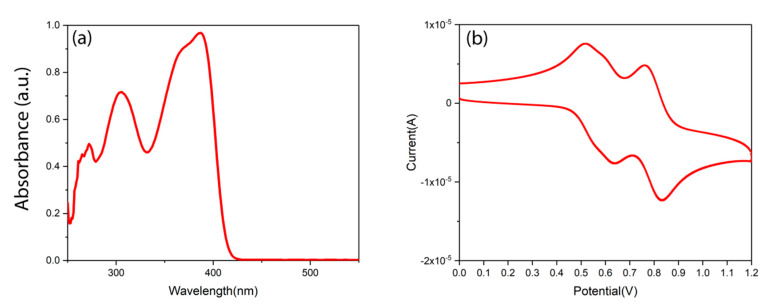
(**a**) Ultraviolet–visible (UV–Vis) spectrum of 2,2′,7,7′-tetrakis[N,N-di(4-methoxyphenyl)amino]-9,9′-spirobifluorene (Spiro) in chlorobenzene solution; (**b**) cyclic voltammetry curve of Spiro in acetonitrile solution.

Small-molecule Spiro is widely used as the HTL in optoelectronic devices. The ultraviolet–visible–near-infrared (UV–Vis–NIR) absorption spectrum of the Spiro in toluene is shown in Figure 2a. The optical bandgap of Spiro is 3.0 eV, calculated from its absorption onset of 412 nm. The electrochemical behavior of the Spiro was investigated by cyclic voltammetry (CV) measurement, which is used to investigate the highest occupied molecular orbital (HOMO) and lowest occupied molecular orbital (LUMO) levels of Spiro. The CV characterization was performed in a solution of Bu4NPF6 (0.1 M) (with acetonitrile as solvent) at a scan rate of 50 mV·s^−1^ at room temperature under argon. A drop of 1 mg·mL^−1^ Spiro/toluene solution was put on the platinum electrode. After drying, the platinum electrode was immersed into the electrochemical cell and used as the working electrode. A Pt wire was used as the counter electrode, while a saturated calomel electrode was selected as the reference electrode. From the CV curve shown in Figure 2b, the oxidation potential (*E*_ox_) is located at 0.80 V. Therefore, the HOMO of Spiro is −5.20 eV according to the empirical formula *E*_HOMO_ = −(*E*_ox_, onset + 4.4), which is confirmed in the literature [27]. The corresponding LUMO level of Spiro is −2.2 eV based on the optical bandgap value and HOMO value (−5.2 + 3.0 = −2.20 eV). In previous work [15], we found that the work function of CdTe NC thin film is 5.17 eV. Therefore, the Spiro will match well with the CdTe NC thin film, which is essential for decreasing energy loss and maximizing output voltage. 

**Figure 3:**
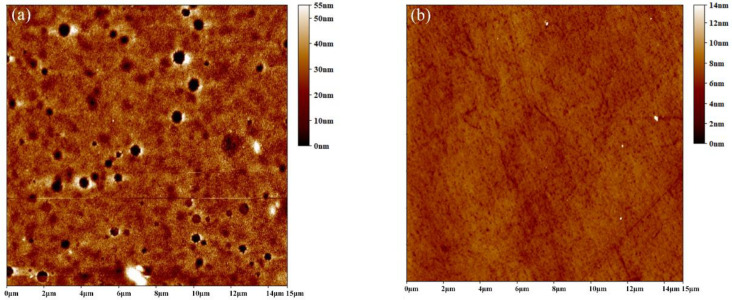
Atomic force microscopy (AFM) images of (**a**) indium tin oxide (ITO)/ZnO/CdS/CdTe and (**b**) ITO/ZnO/CdS/CdTe/Spiro thin films.

Figure 3 shows the top view atomic Atomic force microscopy (AFM) images of as-deposited ITO/ZnO/CdS/CdSe/CdTe/Spiro (with/without) in a large area (15 μm × 15 μm). Although the NC film was compact and flat (with a root mean square value of 7.12 nm), there are still some distinct pinholes along with the continuous CdTe NC film, which may increase the leakage current due to the interface defects (Figure 3a). On the contrary, the NC thin film is covered almost entirely by the Spiro and it shows almost no pinholes in a large area. The whole film is much smoother and more homogeneous with a root mean square value of 4.21 nm. Therefore, the introduction of Spiro HTL between the active layer and Au anode can form a passivation layer on the CdTe NC layer, which is expected to decrease interface recombination and improve carrier collecting efficiencies.

**Figure 4:**
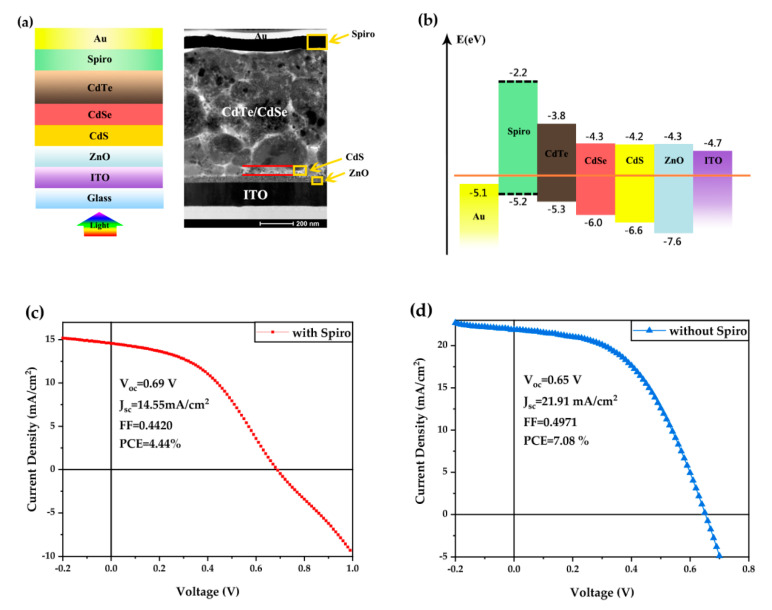
(**a**) The NC solar cell configuration and bright-field (BF) transmission electron microscope (TEM) cross-sectional image of the corresponding device; scale bar: 200 nm; (**b**) band alignment of the NC solar cell with the Spiro hole transfer layer (HTL); (**c**) current density vs voltage (*J–V*) curve of NC solar cells with the Spiro HTL (prepared at room ambient condition) and (**d**) *J–V* curve of the control device (without using the Spiro HTL).

Figure 4a shows the schematic diagram of the NC solar cell with a bright-field (BF) transmission electron microscope (TEM) cross-sectional image of the actual device. The device includes a 40 nm ZnO/ITO cathode, a gradient electron transport layer (30 nm CdS NC/60 nm CdSe), a ~500 nm CdTe NC active layer, and a Spiro-OMe/Au anode. The CdSe_x_Te1−x alloy layer is formed easily when growing and post-treating the CdTe layer as Se has a high miscibility with Te [25]. Therefore, there is no clear boundary between the CdSe NC and CdTe NC layer. The band alignment of the NC device is presented in Figure 4b. The valance band (VB) of CdTe NC is 5.3 eV, which is slightly higher than the HOMO of Spiro HTL (5.2 eV). As Spiro is *p*-type doping, holes can be collected by the anode while the electron can be reflected at the interface of NC/HTL (−2.2 eV). To investigate the effect of Spiro HTL on device performance, solar cells with configurations of ITO/ZnO/CdS/CdSe/CdTe/Spiro (with/without)/Au were fabricated. The devices with the Spiro HTL are preserved in a desiccator for overnight oxidation of Spiro to improve conductivity, which were widely adopted for perovskite solar cells [27]. Figure 4c,d presents the *J*–*V* curves of CdTe NC solar cells with/without HTL, while the insets show the performance parameters such as *V*_oc_, *J*_sc_, FF, and PCE. The control device under optimized conditions (device structure: ITO/ZnO/CdS/CdSe/CdTe/Au) shows a *V*_oc_ of 0.65 V, a *J*_sc_ of 21.91 mA cm^−2^, and a FF of 49.71%, resulting in a PCE of 7.08%, which is similar to the results reported in our previous works [16]. In the case of the NC solar cell with the Spiro HTL (device structure: ITO/ZnO/CdS/CdSe/CdTe/HTL/Au), it shows a high *V*_oc_ of 0.69 V, a very low *J*_sc_ of 14.55 mA cm^−2^, a low FF of 44.2%, leading to a low PCE of 4.44%. As a result, the device performance with HTL is significantly lower than the control device, which is quite different from the previous report [22]. We speculate that since the NC synthesis method and device structure as well as device fabrication process in our research are quite different from the previous report, the amount of interface defects at CdTe/HTL might be at a high level in this device, resulting in large carrier recombination and poor device performance. It should be noted that the *J–V* curve of NC device with HTL shows roll over phenomenon, which is similar to the ones reported by Yang et al. [22]. Large contact resistance at the interface of CdTe/HTL due to the weak interaction between the CdTe NCs and Spiro molecules might account for this phenomenon. 

**Figure 5:**
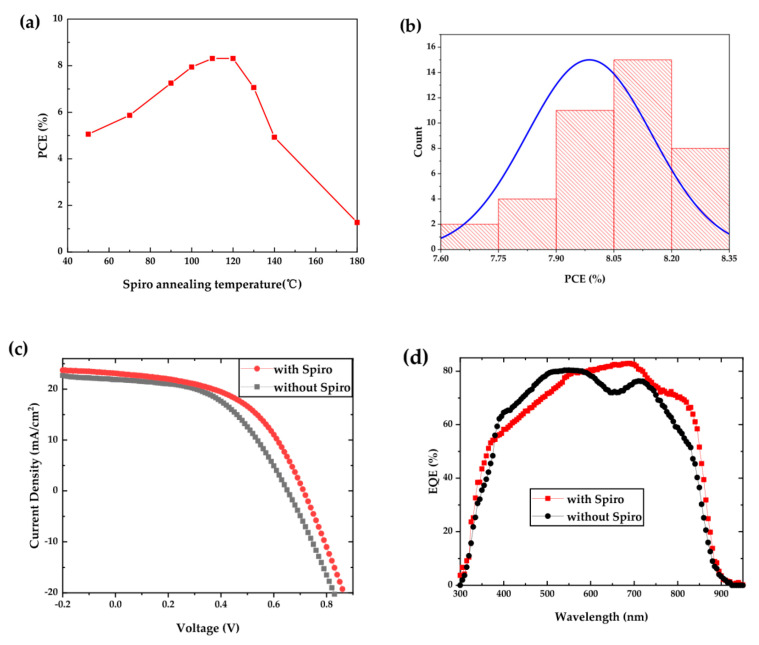
(**a**) The power conversion efficiency (PCE) of NC solar cells with different annealing temperatures; (**b**) the PCE distribution for NC solar cells with the Spiro HTL annealed at 120 °C; (**c**) *J–V* curves of champion and control devices and (**d**) the corresponding external quantum efficiency (EQE) spectra.

It is well known that an appropriate annealing temperature is essential for improving the homogeneous morphology, increasing binding force between the HTL and CdTe NC film, eliminating interface defects, and optimizing carrier mobility of HTL. In previous reports [15,16], we found that heat treatment on HTL at around 120 °C can significantly improve device performance by improving carrier collection efficiency and reducing contact resistance. Inspired by these successful practices, in order to overcome the drawback of contact resistance, the ITO/ZnO/CdS/CdSe/CdTe/HTL samples are heat-treated at different annealing temperatures from 25 to 180 °C for 10 min at ambient conditions. Then they are placed in a desiccator overnight before Au deposition. Figure 5a shows the PCE of NC solar cells with different HTL annealing temperatures, while the *J–V* curves with different annealing temperatures are presented in Figure S1. The photovoltaic parameters are summarized in Table 1. It is found that PCE increases linearly as temperature increases from 50 to 120 °C, which is primarily ascribed to the increasement in *J*_sc_ from 13.04 to ~23 mA/cm^2^. Noted that the optimized annealing temperature is located between 110 and 120 °C. A PCE up to 8% is obtained in these cases, which shows almost two times higher than that of a device with HTL annealed at 50 °C. The NC device performance decays when the annealing temperature further increases and a PCE of only 1.27% is obtained at 180 °C. In addition, NC devices with the Spiro HTL annealed at 110~130 °C show low series resistance (*R*_s_, Table 1) as a result of reduction in interfacial contact resistance (CdTe/HTL). From Figure 5b, one can see that the NC devices have very good repeatability. The 40 devices at least from eight individual batches are fabricated with PCE ranging from 7.6% to 8.4%, producing an average value of about 8.2% (annealing temperature at 110 and 120 °C). More importantly, there is almost no roll over phenomenon on *J–V* curves of devices annealed at temperature higher than 80 °C. Figure 5c,d presents the *J–V* and EQE curves of the champion and control devices. The champion device annealed at 120 °C displays the following figures of merit: *J*_sc_ of 23.11 mA/cm^2^, *V*_oc_ of 0.71 V, FF of 50.83%, and a high PCE of 8.34%, which is among the highest PCE ever reported for CdTe NC solar cells with an inverted configuration. The PCE value obtained here shows a 28% improvement compared to previous report about CdTe NC solar cells with Spiro as HTL [22] (Table 1) and is 18% higher than the control device. From Figure 5d, the EQE spectrum of device with the Spiro HTL shows a higher value than that of the control device from 600 to 900 nm, which attributes to the reduction of interface recombination on CdTe/contact electrode and increase of effective carrier collection. By integrating the EQE curves, the calculated *J*_sc_ of 23.10 mA/cm^2^ and 21.90 mA/cm^2^ are predicted, which are consistent with the measured *J*_sc_ presented in the *J*–*V* curves. The obvious improvement in performance of NC devices with Spiro HTL implies that Spiro is a good hole transfer material for CdTe NC solar cells. Among the devices with HTL, compared to the device without annealing, the device annealed at ~120 °C shows apparently higher PCE (8.34% vs. 4.44%). This might be a result of more effective exciton separation and carrier collection due to the reinforcement of the Cd-N bond and enhancement of the inner electric field by the formation of dipole layer (CdTe/HTL) when annealing at a relatively high temperature. To confirm this, the surface of ITO/ZnO/CdS/CdSe/CdTe/Spiro is characterized by X-ray photoelectron spectroscopy (XPS). From the narrow scan XPS of Cd 3d 3/2 and 5/2 spectrum for Spiro annealed at 120 °C and 25 °C (Figure S2 and Table S1), one can see that the binding energies of Cd 3d5/2 and Cd 3d3/2 are 411.88 eV and 405.08 eV, respectively, for the film annealed at 25 °C, while these values are 412.18 eV and 405.38 eV for Spiro annealed at 120 °C, respectively. Therefore, a higher 0.3 eV value is obtained for Spiro annealed at 120 °C, which implies a strong Cd-N bonding existence in this case, conforming to the results reported before [16]. Moreover, as shown in Figure S3a,b, compared to the HTL without annealing, the HTL annealed at 120 °C presents a smoother surface (root mean square value of 2.56 vs. 4.49 nm), which indicates less interface defects. The decrease in device performance at a relatively high annealing temperature (>130 °C) is likely to be a result of excess diffusion of Li into active layer and excess oxidation of Spiro at high temperature as the annealing treatment was carried out at ambient conditions.

**Figure 6:**
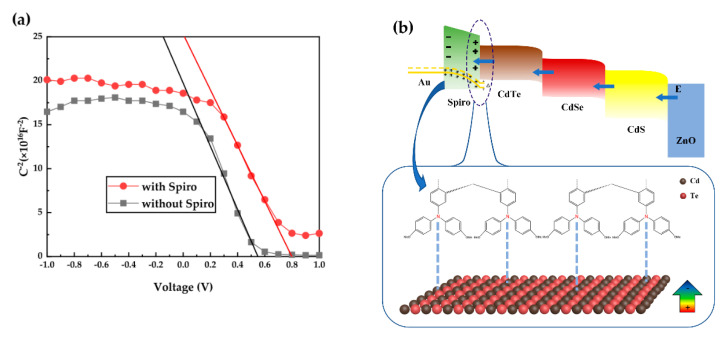
(**a**) Mott–Schottky capacitance−voltage (*C–V*) curves of NC solar cells with/without Spiro; (**b**) a schematic of interfacial dipole and electric field of the NC solar cell with the Spiro HTL.

The *V*_oc_ of CdTe NC solar cells is relatively proportional to the built-in potential (*V*_bi_) generated from the *p*-type CdTe_x_Se_1-x_ alloy and *n*-type CdS NCs. With the appearance of Spiro-OMe HTL, a positive potential between CdTe and HTL is created as a result of the formation of the Cd-N bond, as shown in Figure 6a. It reinforces the *V*_bi_ and usually results in a high *V*_oc_ of the device, like in this case. To provide a solid support for this hypothesis, *C*−*V* analysis with increasing bias voltage at a constant frequency of 1000 Hz was carried out to measure the built-in field of the NC solar cells with/without HTL. Figure 6a shows the plots of *C*^−2^ with bias voltage (*V*). Based on the Mott−Schottky equation [28], the relationship between the capacitance (*C*^−2^) and applied voltage (*V*) is expressed as follows:

(1) $$C^{-2}=\frac{2}{A^{2}q\varepsilon_{0}\varepsilon N_{A}}\left(V_{\mathrm{bi}}-V\right)$$

where *A* is the active area of NC solar cells, *V*_bi_ the built-in potential, *q* is the elementary charge, *ε* is the relative dielectric constant (10.6 for CdTe), *ε*_0_ is the permittivity of vacuum, and *N*_A_ is the net acceptor concentration in an active layer. The value of *V*_bi_ is obtained by fitting a line at a forward bias from the slope and the extrapolated intersection with the x-axis. The *V*_bi_ for NC solar cells with/without HTL is 0.72 V and 0.65 V, respectively, which agrees well with their *V*_oc_ values obtained from the *J–V* curves. Like the TPA and TPP polymer, there are many amine groups that exist in the Spiro molecular framework. The Cd-N bond is formed during the annealing process and a dipole layer can be formed at the CdTe/HTL interface. Similar results have been found in the case of ZnO/oligophenylene interface with a phosphonate-based self-assembled monolayer [29]. Therefore, the direction of the dipole moment is the same as the *V*_bi_ generated from the active layer, which will enhance *V*_bi_ and improve holes transport as well as lower the electron/hole recombination at the CdTe/Au interface, resulting in higher *V*_oc_ in the solar cells with HTL.

**Figure 7:**
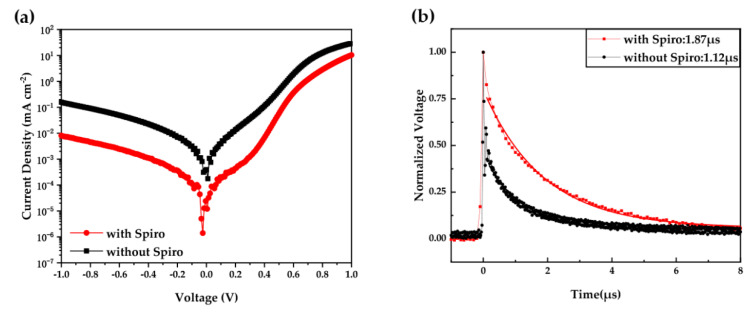
(**a**) *J–V* curves under dark and (**b**) transient photovoltage (TPV) measurements of the champion and control devices.

Compared to the control device, the improvement in NC solar cells with HTL is mainly attributed to the increase in *J*_sc_ and *V*_oc_. To further investigate the effect of HTL on the recombination process of NC solar cells, we measured the *J–V* curves under dark. From the dark *J*–*V* curves (Figure 7a), one can observe that the reverse current in the NC solar cell with HTL was significantly lower than that of the control device. Lower leakage current obtained in the device with HTL implies that the presence of HTL between the CdTe NC layer and Au anode leads to the passivation of interface defects and reduction of carriers’ recombination, resulting in a significant improvement in *J*_sc_ and device performance. Figure 6b shows the interfacial dipole and electric field in the NC solar cells with Spiro as an HTL. The formation of the dipole layer with electric dipole moment points toward the anode. Certainly, the conductivity, mobility of Spiro, and the diffusion of Li into CdTe NC film also affect the device performance. A systematic study of the effects of these factors on the device performance is still ongoing and will be reported in our following work. Here we present the TPV measurement to investigate the charge recombination in NC solar cells with/without the Spiro HTL. In the TPV measurement, the NC solar cells are placed under a white light bias and additional amounts of charges are generated by applying another weak laser pulse. Figure 7b shows the charge recombination of NC devices with/without HTL, which are characterized by tracking the transient voltage associated with the perturbations in charge population. The charge recombination time for the NC device with Spiro HTL is 1.87 μs, while this value of the control device without the Spiro HTL is 1.12 μs. In other words, compared to the control device, a low charge recombination rate exists in the device with Spiro HTL, which is conformed to the higher *J*_sc_ and FF obtained in the device with HTL.

In conclusion, we developed Spiro as the HTL for solution-processed CdTe NC solar cells. It was found that by performing annealing treatment after the deposition of HTL, the improvements in hole mobility and conductivity can be obtained in NC solar cells with Spiro HTL. Simultaneous improvements in *V*_oc_, *J*_sc_, and FF for CdTe NC solar cells are achieved when the annealing temperature is located among the range of 100 to 130 °C. At an optimized annealing temperature of 120 °C, a champion device with a high PCE of 8.34% was obtained, which shows a 28% higher PCE than the NC device with Spiro HTL reported before. The introduction of Spiro can enhance built-in potential across the solar cell owing to interface dipole effect and decrease interface recombination due to increase in *J*_sc_. The research reported here provides a versatile method to further improve CdTe NC solar cells with efficiency towards 10% from various design and material systems. 

## Figures and Tables

**Table 1:** Summarized performance of NC solar cells with the Spiro HTL annealed at different temperatures (Figure 5a) and Literature [22].

Spiro Annealing Temperature (°C)	*V*_oc_ (V)	*J*_sc_ (mA/cm^2^)	FF (%)	PCE (%)	$R_{s}$ $\left(\Omega \cdot cm^{2}\right)$	$R_{sh}$ $\left(\Omega \cdot cm^{2}\right)$
**50**	0.70	13.04	55.43	5.06	13.7	239.5
**70**	0.70	15.61	55.64	6.08	11.5	268.5
**90**	0.66	21.26	51.67	7.25	9.5	455.0
**100**	0.67	23.94	49.38	7.92	8.5	217.0
**110**	0.70	23.75	50.11	8.33	8.8	235.3
**120**	0.71	23.11	50.83	8.34	8.6	229.1
**130**	0.69	18.41	55.66	7.07	9.9	316.8
**140**	0.69	12.19	58.61	4.93	13.0	394.4
**180**	0.61	5.09	40.90	1.27	46.3	273.0
**Literature** [22]	0.71	18.78	49.20	6.56	--	--
**Controlled device**	0.65	21.90	49.71	7.08	10.2	291.6

## Supplementary Materials

The following are available online at https://www.mdpi.com/2079-4991/10/7/1348/s1, Figure S1: *J–V* curves of NC solar cells with HTL annealed at different temperatures; Figure S2: The narrow XPS spectra of Cd3d for ITO/ZnO/CdS/CdSe/CdTe/Spiro with different annealing temperature; Table S1: Summarized XPS values of Cd3d with HTL annealing at different temperatures (Figure S2); Figure S3: AFM images of HTL (a) without annealing (25 °C) and (b) with annealing at 120 °C.
