# Supplementary material for: Hole Transfer Layer Engineering for CdTe Nanocrystal Photovoltaics with Improved Efficiency
Source: Nanomaterials (Basel). 2020 Jul 10;10(7):1348. doi: 10.3390/nano10071348 (PMC7407640; doi:10.3390/nano10071348)
Supplement: Supplementary file 1 [file nanomaterials-10-01348-s001.pdf]

nanomaterials

Supporting information

Hole transfer layer engineering for CdTe nanocrystal photovoltaics with improved efficiency

Yasi Jiang [1,†], Yiyang Pan [1,†], Wanhua Wu [1,†], Kaiying Luo [1,†], Zhitao Rong [1,†], Sihang Xie [1], Wencai Zuo [1], Jingya Yu [1], Ruibo Zhang [1], Donghuan Qin [1,3,*](✉), Wei Xu [1,3], Dan Wang [1,3] and Lintao Hou [2,*](✉)

[1]*Affiliation;School of Materials Science and Engineering, South China University of Technology, Guangzhou 510640, China;*
[2]*Affiliation;Siyuan Laboratory, Guangzhou Key Laboratory of Vacuum Coating Technologies and New Energy Materials, Guangdong Provincial Key Laboratory of Optical Fiber Sensing and Communications, Department of Physics, Jinan University, Guangzhou 510632, China;*
[3]*Affiliation;Institute of Polymer Optoelectronic Materials & Devices, State Key Laboratory of Luminescent Materials & Devices, South China University of Technology, Guangzhou 510640, China;*
*Address correspondence to qindh@scut.edu.cn

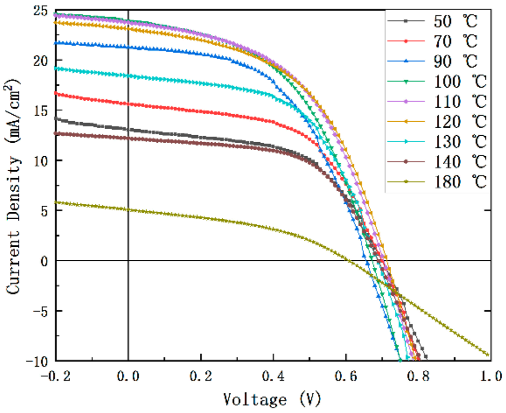

Figure S1. J-V curves of NC solar cells with Spiro HTL annealing at different temperature

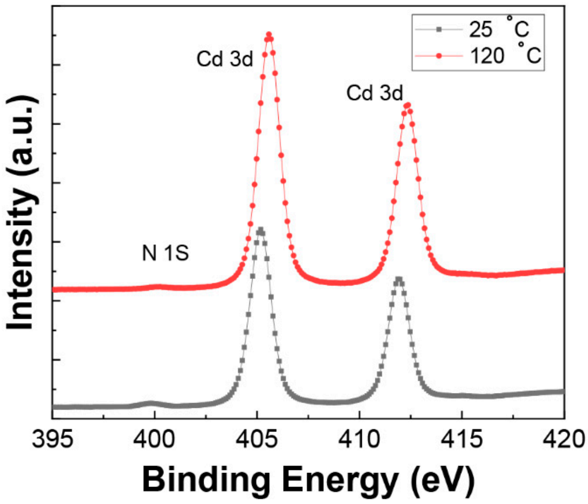

Figure S2. The narrow XPS spectra of Cd3d for ITO/ZnO/CdS/CdSe/CdTe/Spiro with different annealing temperature

Table S1. Summarized XPS values of Cd 3d with HTL annealing at different temperature (Figure S2)

**treatment condition**	**Cd3d5/2**	**Cd3d3/2**
**25 ˚C**	405.08	411.88
**120 ˚C**	405.38	412.18

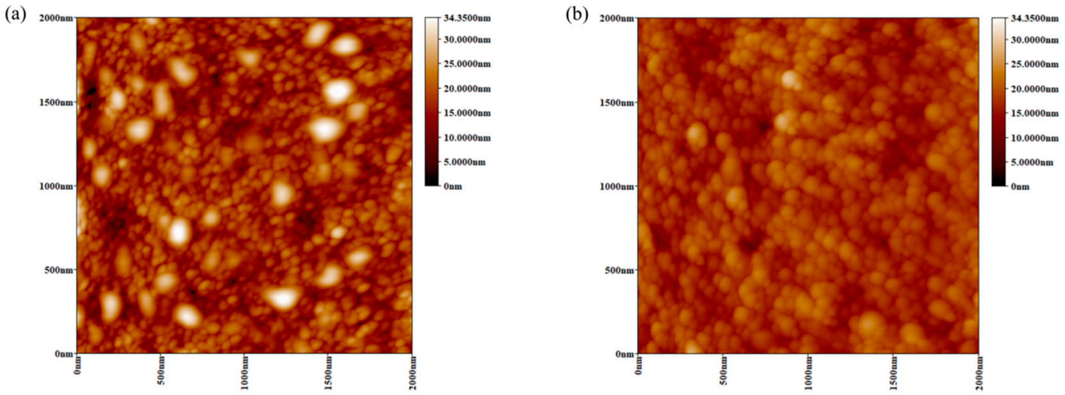

Figure S3. AFM image of HTL (**a**) without annealing (25℃) and (**b**) annealing at 120℃
